# Supplementary material for: Reversal of Right Ventricular Hypertrophy and Dysfunction by Prostacyclin in a Rat Model of Severe Pulmonary Arterial Hypertension
Source: Int J Mol Sci. 2022 May 12;23(10):5426. doi: 10.3390/ijms23105426 (PMC9141343; doi:10.3390/ijms23105426)
Supplement: Supplementary file 1 [file ijms-23-05426-s001.zip › ijms-1707566-supplementary.pdf]

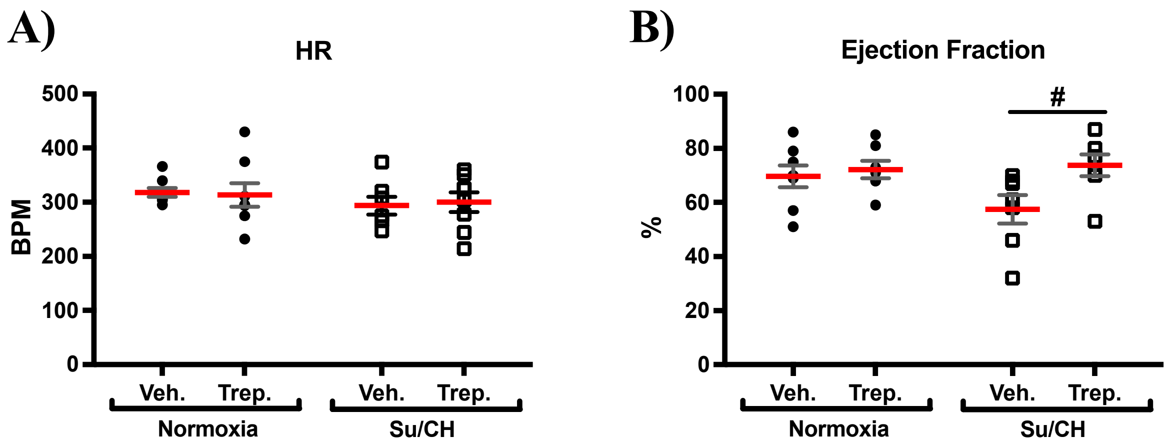

Figure S1. RV function parameters upon SuCH and treprostinil treatment. Individual value grouped plots of (A) heart rate (HR) and (B) RV ejection fraction (EF); n=7-8 animals/experimental group; *, $ and # present significance vs. SuCH+Veh.; # P ≤ 0.05.

**List of RNA array genes**									
Cdca5	Cdkn1a	Cdkn1b	Zeb1	Col1a1	Col1a2	Col3a1	Eif4e	Rps6kb1	Akt2
Dnm1	Slc9a3r1	Esr1	Akt1	Fas	Faslg	Fis1	Fn1	Grb2	Gak3b
Hes1	Hey1	Hey2	Hlf1a	Camk2a	Icam1	Mylk	IL10	IL13	IL13ra2
IL1a	IL1b	IL4	Rheb	IL6	IL6r	Nppb	Nppa	Lats1	Lats2
Lpl	Snai3	Snai2	Mapk1	Mapk8	Myc	Prkaa1	Nf2	Slc9a1	Slc9a3
Notch1	Nox1	Cybb	Nox4	Ntrk1	ulk1	Ulk2	Atg7	Nsfl1c	Pcna
Tfeb	Pparg	Prkaca	Snai1	Ryr2	Scn5a	Serpine1	Sp1	Src	Srebf1
Rhoa	Taz	Tbx20	Tgfa	Tgfb1	Tgfb2	Tgfb3	Tgfbr1	Tgfbr2	Colg4a1
Tle3	Tle4	Tnf	Txn1	Ucp1	Ucp2	Ucp3	Vegfa	Ccn4	Wnt1
Wnt2b	Wnt3a	Wnt5a	Wnt6	Yap1	Ywhaz	Slc9a3r2	Prkca	Actb	gapdh
hsp90ab1									

Figure S2. List of genes included in our custom RNA array.
